# The Role of Alarmins in the Pathogenesis of Atherosclerosis and Myocardial Infarction

**DOI:** 10.3390/cimb46080532

**Published:** 2024-08-17

**Authors:** Kajetan Kiełbowski, Patryk Skórka, Paulina Plewa, Estera Bakinowska, Andrzej Pawlik

**Affiliations:** 1 Department of Physiology, Pomeranian Medical University, 70-111 Szczecin, Poland; kajetan.kielbowski@onet.pl (K.K.); p.skorka04@gmail.com (P.S.); esterabakinowska@gmail.com (E.B.); 2 Institute of Biology, University of Szczecin, 71-412 Szczecin, Poland; paulina.plewa@op.pl

**Keywords:** atherosclerosis, myocardial infarction, alarmins, high-mobility group box 1, S100 proteins, interleukin-33

## Abstract

Atherosclerosis is a condition that is associated with lipid accumulation in the arterial intima. Consequently, the enlarging lesion, which is also known as an atherosclerotic plaque, may close the blood vessel lumen, thus leading to organ ischaemia. Furthermore, the plaque may rupture and initiate the formation of a thrombus, which can cause acute ischaemia. Atherosclerosis is a background pathological condition that can eventually lead to major cardiovascular diseases such as acute coronary syndrome or ischaemic stroke. The disorder is associated with an altered profile of alarmins, stress response molecules that are secreted due to cell injury or death and that induce inflammatory responses. High-mobility group box 1 (HMGB1), S100 proteins, interleukin-33, and heat shock proteins (HSPs) also affect the behaviour of endothelial cells and vascular smooth muscle cells (VSMCs). Thus, alarmins control the inflammatory responses of endothelial cells and proliferation of VSMCs, two important processes implicated in the pathogenesis of atherosclerosis. In this review, we will discuss the role of alarmins in the pathophysiology of atherosclerosis and myocardial infarction.

## 1. Introduction

The term atherosclerosis describes the condition of accumulating fat and fibrous tissue in the arterial intima layer. Atherosclerosis is a major background condition that leads to the development of cardiovascular diseases (CVDs). Atherosclerotic lesions, also known as plaques, tend to develop and increase in size as the disease progresses, which can eventually alter blood flow and lead to tissue ischaemia. Furthermore, atherosclerotic plaques can promote the development of thrombi, which can close the lumen and manifest as acute ischaemia. Plaques developing in coronary arteries can cause acute coronary syndromes, whereas those developing in carotid arteries cause can ischaemic strokes [1]. Atherosclerosis is a condition with a large global burden; a meta-analysis of epidemiological data from 2020 demonstrated that 27.6% of people aged 30 to 79 years had an increased carotid intima-to-media thickness, whereas the prevalence of carotid plaques was estimated at 21.1% [2]. The pathogenesis of atherosclerosis involves interactions between different cell populations, secreted cytokines, chemokines, and other mediators, in addition to lipoprotein modification and accumulation. Among immune cells, interactions between macrophages, dendritic cells, and T cells induce an inflammatory environment that contributes to the progression of atherosclerotic plaques [3]. Furthermore, endothelial dysfunction, foam cell formation, and vascular smooth muscle cells (VSMCs) are key factors that are implicated in the accumulation of fat and fibrous tissue in the arteries. Numerous VSMC phenotypes are present in atherosclerotic plaques, and Chen et al. have published a comprehensive review summarising the characteristics of these cells [4]. Low-density lipoproteins (LDLs) transport cholesterol to target cells in the vasculature, and they are strongly associated with atherosclerosis. LDL molecules undergo modifications that play a role in the progression of atherosclerosis; one of the most investigated forms of modified LDL is oxidised LDL (ox-LDL), and this modification is considered to occur in the vascular wall. Importantly, other atherogenic modifications are also implicated in lipid accumulation [5]. Due to its extremely high prevalence the potentially severe complications it comes with, it is crucial to understand the complex pathological mechanisms that are involved in the progression of atherosclerosis in order to develop novel treatment methods to prevent major adverse cardiological events.

Alarmins are a broad family of proteins that can activate immune responses. They can be released due to cellular death or injury, or as a result of immune induction. As they interact with pattern recognition receptors (PRRs) such as Toll-like receptors (TLRs), alarmins are frequently called damage-associated molecular patterns (DAMPs) [6]. Interestingly, they can also stimulate adaptive immune responses [6]. Various proteins belong to the family of alarmins, but some of the more investigated members include high-mobility group box 1 (HMGB1), S100 proteins, interleukin 33 (IL-33), and heat shock proteins (HSPs), among others. HMGB1 has different functions depending on its subcellular and extracellular location. Post-translational modifications determine the location of HMGB1, which can be either in the nucleus, where it can bind chromatin, or in the cytoplasm, from which it can be released into the extracellular space, where it acts as a DAMP [7]. HMGB1 mediates inflammatory responses by interacting with, and signalling through, TLR4, as well as the receptor for advanced glycation end products (RAGE) [8]. Among the S100 proteins, S100A8 and S100A9 seem to be those most investigated; they form a heterodimer, S100A8/A9, that is also known as calprotectin. S100A8/A9 also interacts with the TLR4 receptor, thereby inducing pro-inflammatory responses [9]. IL-33 belongs to the IL-1 cytokine family, which interacts with, and signals through, the ST2 receptor to regulate innate and adaptive immunity in addition to modulating the activity of several signalling pathways [10]. Dysregulated expression of alarmins has been observed in patients with inflammatory diseases, which, considering their immunoregulatory properties, could be implicated in the pathogenesis of these diseases. In our previous work, we discussed the involvement of alarmins in the mechanisms associated with rheumatoid arthritis, osteoarthritis, and psoriasis [11]. The aim of this article is to summarise current evidence that HMGB1, S100 proteins, and IL-33 play roles in the pathogenesis of atherosclerosis and myocardial infarction.

## 2. Alarmins and Atherosclerosis

### 2.1. HMGB1

To begin with, levels of circulating HMGB1 are elevated in patents with hyperlipidaemia, which is a state that is significantly is associated with the development of atherosclerosis, but treatment with atorvastatin reduces HMGB1 concentrations [12]. High serum levels of HMGB1 have also been observed in non-diabetic patients with CAD [13]; monitoring of HMGB1 could indirectly aid in discriminating plaque characteristics, as this could allow us to detect non-calcified plaques [14]. Higher expression of HMGB1 has been observed in peripheral blood mononuclear cells (PBMCs) in patients with coronary atherosclerosis compared with patients with normal coronary arteries [15]. Serum levels of HMGB1 are also positively correlated with IL-6 levels in patients with CAD [16]. Therefore, current evidence demonstrates the greater presence of HMGB1 in patients with atherosclerosis or in conditions that lead to the progression of the vascular disorder. Taking into consideration the pro-inflammatory nature of HMGB1, these findings could indicate that HMGB is strongly involved in the background mechanisms driving the progression of atherosclerosis. In rats with diabetes mellitus and atherosclerosis, treatment with the HMGB1 inhibitor gycyrrhizic acid was able to reduce the levels of pro-inflammatory mediators in the serum and aortas of animals [17]. Thus, it is important to discuss how alarmin might affect cells and tissues actively taking part in the progression of atherosclerosis.

Studies have demonstrated that HMGB1 affects the behaviour of various cells associated with atherosclerosis, including VSMCs, endothelial cells, macrophages, and T cells. Firstly, alarmin is expressed in atherosclerotic plaques. In an early study performed by Inoue et al., HMGB1 immunoexpression was detected in the intima of plaques from carotid endarterectomy samples. Furthermore, the authors detected alarmin in both macrophages and VSMCs; these same cell populations were found to be positive for HMGB1 in samples of coronary atherosclerosis. Interestingly, the authors also performed an in vitro experiment investigating the influences of alarmin on pro-inflammatory responses by VSMCs. Stimulation of VSMCs with HMGB1 increased the expression of mRNAs for C-reactive protein (CRP) and matrix metalloproteinase 2 (MMP-2) [18]. These results suggested that HMGB1 participates in inflammatory responses and affects plaque vulnerability, as MMP-2 is thought to participate in the progression of atherosclerosis in multiple ways [19]. The relationship between HMGB1 and inflammation is also complex and involves several mechanisms. Pretreatment of VSMCs with IL-1β has been shown to enhance the expression of both HMGB1 and its receptor, RAGE; this upregulation involves the P2Y_2_R receptor and the ROCK2, Rac-1, AKT, ERK, and PKC pathways [20]. In turn, HMGB1 enhanced the production of IL-1β in VSMCs, which is dependent on the TLR2, TLR4, and RAGE receptors. Furthermore, alarmin stimulated pro-inflammatory responses through the inflammasome of NLR family pyrin domain-containing proteins 3 (NLRP3) [21]. NLRP3 is a major pro-inflammatory structure that enhances pyroptosis, which is a mechanism of cellular death (a detailed description can be found in [22]). Importantly, several studies have demonstrated that NLRP3 is a potential treatment target in atherosclerosis, as it is involved in pathways that enhance the progression of the disease but are suppressed by NLRP3 inhibitors [23,24]. Studies suggest that NLRP3 and HMGB1 could be correlated in a signalling loop that eventually enhances the formation of atherosclerotic plaques. Specifically, NLRP3 was found to stimulate the translocation of HMGB1 from the nucleus to the cytoplasm, which was followed by its extracellular release by VSMCs. HMGB1 subsequently contributed to cholesterol accumulation in VSMCs in addition to suppressing the expression of LXRα and ABCA1 [25]. Moreover, treatment of VSMCs with HMGB1 was demonstrated to enhance IL-6 production, which was dependent on the p38 and ERK1/2 signalling pathways [16].

**Figure 1:**
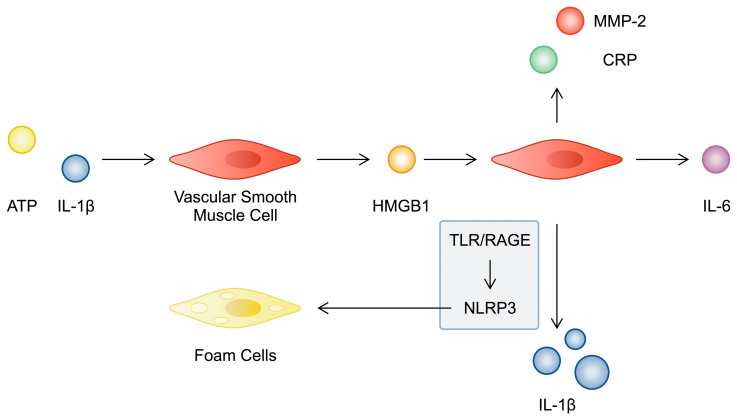
A schematic illustration depicting the modulation of VSMC behaviour by HMGB1, which is correlated with inflammation and atherosclerosis pathogenesis.

Ox-LDLs are major molecules involved in the pathogenesis of atherosclerosis. VSMCs treated with ox-LDLs change their phenotype from contractile to synthetic, which is associated with the migration and proliferation of these cells. Intriguingly, apart from reversing the abovementioned modifications, HMGB1 silencing can also potentiate the anti-inflammatory properties of other agents. In a study by Hu et al., the authors demonstrated that the use of SiHMGB1 boosted the beneficial effects of sodium ferulate, which is a component of Chinese medicines [26]. Importantly, drugs used in the treatment of patients with CVDs induce pleiotropic effects. Yeh and colleagues showed that labedipinedilol-A and lercanidipine, which are dihydropyridine calcium channel blockers could suppress the production of reactive oxygen species (ROS), TNFα, and MMPs in addition to HMGB1 in rat VSMCs [27]. Thus, several lines of evidence indicate the involvement of HMGB1 in pathological responses of VSMCs that are correlated with atherosclerosis (Figure 1). In a recent study by Zhang and collaborators, the authors also proved that HMGB1 takes part in vascular calcification, another feature that is typically associated with atherosclerosis. Specifically, the authors observed that liver kinase B1 (LKB1) could bind to and suppress HMGB1, which inhibited the calcification process [28].

The involvement of HMGB1 in pro-inflammatory responses of VSMCs was also investigated in several studies involving non-coding RNAs (ncRNAs). These molecules are major regulators of gene expression, the dysregulation of which has been frequently noted in numerous diseases. MicroRNAs (miRNAs), long non-coding RNAs (lncRNAs), and circular RNAs (circRNAs) are among the most investigated members of the ncRNA family that epigenetically controls gene expression. Altered expression profiles of ncRNAs thus change the expression of inflammatory regulators and signalling pathways, which contributes to disease pathogenesis [29,30]. Importantly, numerous ncRNAs regulate the expression of HMGB1, and several studies have investigated these signalling axes in the context of atherosclerosis and VSMCs. For instance, stimulation of VSMCs with ox-LDL reduces the expression of miR-141-5p. Mechanistically, the molecule has been found to suppress HMGB1 expression and NF-κB activity [31]. Similarly, Jiang et al. showed that the treatment of A7r5 cells with ox-LDL was associated with reduced miR-129-5p expression and enhanced HMGB1 expression. At the same time, ox-LDL stimulation enhanced the migration and viability of treated cells, which were significantly suppressed following transfection with miR-129-5p mimics [32]. Moreover, HMGB1 is targeted by miR-34c and miR-24, which could suppress pro-inflammatory atherogenic responses [33,34]. Intriguingly, the expression and functions of miRNAs are regulated by other members of the ncRNA family, namely lncRNA and circRNA. Jiang and colleagues found an interaction between lncRNA BANCR and miR-34c; in the plasma of patients with atherosclerosis, the expressions of BANCR and miR-34c were increased and decreased, respectively. An in vitro study using human aortic smooth muscle cells revealed that the overexpression of BANCR downregulated the expression of miR-34c. Mechanistically, the authors demonstrated that lncRNA BANCR mediated the methylation of miR-34c and consequently affected the expression of downstream targets of miR-34c; for example, BANCR was able to elevate the expression of HMGB1 [35]. The HOXA transcript at the distal tip (HOTTIP) is another lncRNA that is being investigated in the context of atherosclerosis. Its expression was found to be elevated in atherosclerotic patients and in aortic VSMCs stimulated with ox-LDL. Mechanistically, HOTTIP regulated the miR-490-3p/HMGB1 axis. Thus, lncRNA enhanced VSMC migration and production of MMPs [36]. Similarly, ox-LDL was shown to enhance the expression of circ_0010283, which also regulated the expression of HMGB1 through interacting with miRNA [37]. As ncRNAs are altered in atherosclerosis, the question arises as to whether we could regulate their expression or activities to induce beneficial effects and restore inflammatory homeostasis. Drugs that modulate ncRNA functionality are not a novel idea; clinical trials have been performed to investigate the efficacy and safety of miravirsen, an antisense oligonucleotide with a sequence that is complementary to miR-122. Miravirsen can sequester miR-122, and it has been tested in patients with HCV infection [38]. Other agents and substances have also been found to regulate miRNA expression. These off-target effects could be implemented in clinical practice to induce several beneficial effects. Regarding atherosclerosis, Chen et al. demonstrated that baicalein, a flavonoid compound, could upregulate miR-126-5p and inhibit the expression of HMGB1 in VSMCs stimulated with ox-LDL [39].

As previously mentioned, HMGB1 signals through the RAGE receptor. An early study by Harja and collaborators demonstrated that RAGE-deficient ApoE^−/−^ mice showed reduced markers of vascular inflammation in the aorta, which indicates the involvement of RAGE in the pathogenesis of atherosclerosis [40]. Several studies have also investigated the roles of RAGE in VSMCs in the context of atherosclerosis. In ApoE^−/−^ diabetic mice, pioglitazone was able to suppress the development of atherosclerosis plaques. In cultured VSMCs, pioglitazone was found to reduce the overexpression of RAGE that had been induced by high-glucose conditions [41]. The RAGE receptor contributes to modifying the phenotype of VSMCs, and it has been hypothesised to take part in the transformation of VSMCs into a macrophage-like phenotype [42]. Furthermore, the receptor is involved in foam cell formation by mediating the uptake of modified LDL molecules. Enzymatically modified LDL (EMLDL) has been shown to enhance the expression of LOX-1 in SMCs. However, in RAGE-deficient cells, such stimulation could not induce the expression of the scavenger receptor. According to Chellan et al., RAGE is not considered an ELDL receptor, but it influences the uptake, acting as signalling molecule [43]. A soluble form of the RAGE receptor exists (sRAGE) that can induce beneficial effects by sequestering RAGE ligands and suppressing the classic signalling pathway. In an in vitro experiment involving human aortic smooth muscle cells, angiotensin II could enhance RAGE release and enhance the calcification of the cells that were examined. Vascular calcification is an important process that is mediated by VSMCs and increases CVD risk. Importantly, the introduction of sRAGE inhibited RAGE signalling and attenuated calcium deposition [44,45]. Thus, a significant number of studies have investigated the role of HMGB1 in mediating the activities of VSMCs that could be associated with the development of atherosclerosis. Nevertheless, other cell types were also examined.

Atherosclerosis is highly correlated with endothelial dysfunction. The endothelium is the key element that regulates blood vessel wall homeostasis, and dysfunction in the endothelium is associated with impaired vasodilation together with various prothrombotic and pro-inflammatory properties [46,47]. Intriguingly, HMGB1 is thought to be involved in ox-LDL-mediated endothelial injury. Specifically, Huo et al. demonstrated that stimulation of HUVECs with ox-LDLs enhanced the expression of HMGB1 and promoted its cytoplasmic translocation. The silencing of HMGB1 was able to prevent ox-LDL-induced damage to endothelial cells. Mechanistically, these findings were mediated by the PI3K/AKT pathway [48]. In another study, HMGB1 was found to be a major regulator of LDL transcytosis in human coronary artery endothelial cells (HCAECs). HMGB1 knockdown reduced the expression of sterol regulatory element-binding protein 2 (SREBP2), which is a transcription factor that is implicated in the expression of cholesterologenic genes [49]. Importantly, agents and molecules that alleviate atherosclerosis were also found to downregulate the expression of SREBP2 [50,51,52]. Another phenomenon that is regulated by HMGB1 and implicated in the pathogenesis of atherosclerosis is that of endoplasmic reticulum (ER) stress. ER stress is associated with the development of misfolded proteins, which induces inflammatory responses and apoptosis. Kim and Woo demonstrated that, compared with endothelial cells stimulated with ER stress inducers, laminar flow could inhibit cellular apoptosis. Thus, anti-inflammatory mechanisms known to have anti-atherogenic potential can overcome cellular damage induced by ER stress [53]. The HMGB1/RAGE axis was found to contribute to endothelial ER stress and inflammation [54]. Similar to the HMGB1/NLRP3 interaction demonstrated in VSMCs, the HMGB1/RAGE/NLRP3 pathway also affected the behaviour of endothelial cells: suppression of this axis prevented endothelial cell apoptosis. Interestingly, the expression of HMGB1 was enhanced due to chronic intermittent hypoxia, a process that is associated with sleep apnoea and the development of atherosclerosis [55]. As previously mentioned, sRAGE sequesters RAGE ligands, which is associated with beneficial outcomes. In the endothelium, the activity of sRAGE suppresses increased permeability, which confirms its positive effects [56]. Table 1 summarises selected mechanisms that link HMGB1 with VSMC and endothelial cells and are associated with atherosclerosis.

### 2.2. S100 Proteins

In patients with CAD and diabetes, serum levels of S100A8/A9 are elevated and positively associated with the severity of the disease [57]. S100A8/A9 is strongly associated with neutrophils, and plasma levels of the alarmin complex are enhanced by these circulating cells [58]. In atherosclerotic lesions, neutrophils are thought to enhance inflammatory responses and stimulate endothelial permeability. Moreover, as these cells contain enzymes that degrade components of the extracellular matrix (ECM), they can contribute to fibrous cap weakening and plaque rupture [59], thereby driving the formation of thrombi and the subsequent development of acute ischaemia. Importantly, compared with those of patients with stable disease, higher serum concentrations of S100A8/A9 were noted in patients with unstable angina. In experiments analysing atherectomy specimens, a higher expression of S100A8/A9 was observed in tissues from patients with unstable disease; the majority of the S100A8/A9-positive cells detected in this study were neutrophils [60]. Apart from weakening the fibrous cap, neutrophils exert another important activity implicated in the pathogenesis of atherosclerosis: these cells release neutrophil extracellular traps (NETs), which contain antimicrobial proteins, histones, and DNA. NETs have been found in atherosclerotic plaques, as well as in thrombi in the coronary arteries [61]. The release of NETs (NETosis) stimulates pro-inflammatory responses, which is thought to contribute to endothelial dysfunction [62]. It has been suggested that, during NETosis, S100A8/A9 is also released from neutrophils and that, in turn, the alarmins can activate neutrophils and stimulate their adhesion [63]. Another molecule that affects vascular physiology is S100A12. Transgenic mice expressing S100A12 demonstrated thickened aorta walls, enhanced collagen deposition, and upregulated MMP-2 expression. Furthermore, alarmin induced IL-6 expression and oxidative stress [64]. In atherosclerosis-prone ApoE^−/−^ mice, S100A12 was shown to enhance calcification of the aorta. In addition, under inflammatory conditions, alarmin stimulated an osteoblastic profile of VSMCs in these mice [65]. Indirect atherogenic properties of S100A12 relating to macrophages have also been observed. Specifically, S100A12 upregulated IL-22 through the RAGE receptor, which then inhibited cholesterol efflux from macrophages, suggesting that S100A12 might contribute to foam cell formation [66]. S100 proteins seem to be also associated with metabolic alterations observed in atherosclerosis. Glycolysis has been associated with pro-inflammatory macrophage polarisation [67]. Inhibition of glycolysis has been recently correlated with reduced expression of S100A8 and S100A12 [68].

### 2.3. Interleukin-33

The involvement of IL-33 in the pathogenesis of atherosclerosis remains controversial, as conflicting results have been published. According to Stankovic et al., elevated serum levels of IL-33 were observed in patients with carotid atherosclerosis. Moreover, the expressions of IL-33 and its receptor ST-2 were abundant in various types of plaques. However, fibrous plaques showed higher expression of IL-33 and ST-2 compared with vulnerable lesions. The dysregulated expression of IL-33 suggests that it might be somehow involved in the pathogenesis of atherosclerosis. The authors also demonstrated that IL-33 was associated with a more inflammatory profile in atherosclerotic lesions [69]. Therefore, this study suggested that IL-33 might induce pro-inflammatory and pro-atherogenic effects. However, other studies showed that IL-33 signalling attenuated the progression of this arterial condition. For example, the introduction of IL-33 to ApoE^−/−^ mice fed with a high-fat diet reduced atherosclerotic lesions. Conversely, the introduction of soluble ST2 receptor (sST2) was associated with significant disease progression [70]. 

### 2.4. Heat Shock Proteins

Heat shock proteins are a family of proteins that are upregulated in response to stressful conditions. The family involves several members, including small HSP (sHSP), HSP40, HSP60, and HSP90, among others. By acting as chaperones, these proteins improve damaged proteins. HSPs prevent unfolded protein aggregation. Moreover, they enhance the degradation of misfolded proteins through proteases or the process of autophagy [71,72]. In recent years, several studies have investigated the potential involvement of HSPs in the pathogenesis of atherosclerosis.

Beginning with HSP60, genetic variants encoding the protein are associated with advanced atherosclerosis [73]. Previously, it has been suggested that HSP60 can enhance the progression of this disorder. Lately, researchers are investigating whether antigenicity of HSP60 could be used to protect patients from developing atherosclerosis. Specifically, potential immunisation with HSP60 has been evaluated. Subcutaneous administration of recombinant HSP60 reduces plaque formation in rabbits [74]. In another study, Hu et al. examined if the administration routes could be associated with different results. Intriguingly, researchers observed that in ApoE^−/−^ mice fed a Western diet, subcutaneous administration was associated with increased plaque size. In contrast, an oral route of HSP60 decreased plaque size. Mechanistically, oral HSP60 administration induced beneficial immunological effects. The authors observed that it reduced the levels of IFN-γ and increased those of IL-10. These mechanisms could be related to the expansion of myeloid-derived suppressor cells (MDSCs) [75]. MDSCs seem to play a protective role in atherosclerosis, which was recently summarised in a paper by Li and collaborators [76]. Hypothetically, the beneficial effects of oral HSP60 could be explained with the previously described effect of mucosal tolerance [77]. However, the results suggesting the involvement of subcutaneous administration of HSP60 in the pathogenesis of atherosclerosis make its role more complex. Perhaps, responses induced by HSP60 depend on the cellular context or the model of cells and animal species. Recently, researchers demonstrated that autoimmunity targeting HSP60 further drives the progression of atherosclerotic plaques. Introduction of serum obtained from HSP60 immunised mice into ApoE^−/−^ mice with induced atherosclerosis significantly increased plaque progression. Moreover, serum administration was associated with greater infiltration with M1 macrophages, suggesting an enhanced inflammatory response [78].

Several lines of evidence suggest the involvement of HSP60 in the pathogenesis of atherosclerosis. Specifically, the protein mediates the detrimental effects of oxLDL. Knockdown of HSP60 prevents reduced production of nitric oxide in HUVECs. Moreover, HSP60 deficiency inhibits monocyte adhesion to endothelial cells. Thus, HSP60 is considered to play a significant role in endothelial dysfunction, which is a crucial background mechanism associated with vascular disease. Interestingly, HSP60 knockdown also affects macrophages. A deficiency of stress response protein was associated with the elevated accumulation of oxLDL and increased polarisation towards the pro-inflammatory M1 phenotype. These results indicate the various effects of HSP60 depending on cell types [79]. Perhaps, the protein might also induce different cellular responses depending on the environment. For instance, THP-1 monocytes secrete HSP60 under hyperglycemic conditions. Subsequently, the secreted protein can induce pro-inflammatory responses in HUVECs. These results were not observed when cells were stimulated with reduced concentrations of glucose [80]. Additionally, HSP60 indirectly activates T cells, another major cellular component of plaques. Several subtypes of T cells contribute to the progression of atherosclerosis, which was comprehensively summarised in a recent review article [81]. HSP60 influences the behaviour of T cells through dendritic cells (DCs). Rahman et al. analysed the impact of HSP60 on DC maturation. Researchers observed that co-culture of stimulated DCs with T cells enhanced the production of pro-inflammatory cytokines, together with the expression of transcription factors associated with Th1, Th17, and Treg cells [82]. The stress response molecule also affects VSMCs by enhancing their proliferation [83].

**Figure 2:**
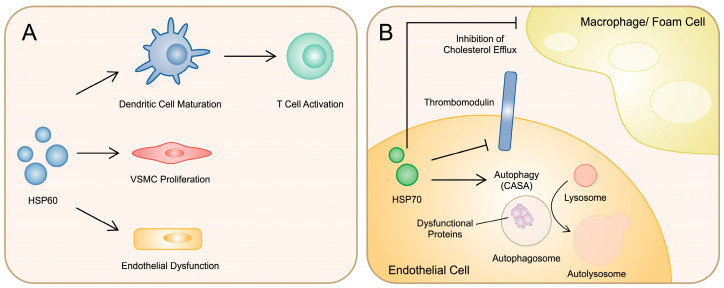
Summary of potential mechanisms induced by HSPs associated with atherosclerosis. (**A**) HSP60 influencing endothelial cells, vascular smooth muscle cells, and dendritic cells to induce atherogenic responses. (**B**) HSP70 negatively regulating thrombomodulin and taking part in the process of autophagy in endothelial cells, as well as suppressing cholesterol efflux from macrophages.

HSP70 is involved in regulating several crucial cellular mechanisms, such as protein folding, translocation, and disaggregation [84]. As it can induce both pro- and anti-inflammatory responses, it is generally considered protective under stressful conditions. However, HSP70 was suggested to participate in the pathophysiology of CVDs. In endothelial cells, a pro-atherogenic flow reduces the expression of HSP70. Mechanistically, the protein binds to and negatively regulates the activity of thrombomodulin, which plays a protective role in atherosclerosis [85]. Thus, downregulation of HSP70 could then increase the positive influence of thrombomodulin and overall suppress pro-atherogenic responses [86]. In a more direct study, researchers evaluated the influence of HSP70 overexpression on atherosclerotic lesion formation in ApoE^−/−^ mice. The authors observed that increased expression of stress response protein was associated with lipid lesions of a greater size and a greater number of them. HSP70 was found to suppress cholesterol efflux by reducing the expression of ABCA1 and ABCG1 [87]. Hypothetically, HSP70 could exert various effects depending on the cell type or atherogenic model. Furthermore, endothelial cells could have protective mechanisms to respond to HSP70, which might not be present in macrophages. The ability of the endothelium to use HSP70 in an anti-atherogenic mechanism has been confirmed in another study. HSP70 is involved in a complex known as chaperone-assisted selective autophagy (CASA). Autophagy is a major cellular process responsible for the degradation of cellular compartments to recycle them and maintain homeostasis. CASA can degrade misfolded and damaged proteins [88]. In atherosclerosis, it is considered that dysfunctional autophagy is correlated with endothelial dysfunction. B cell lymphoma 2-associated athanogene (BAG3) is a crucial element of the CASA complex that binds to HSPs. Diao and collaborators demonstrated that overexpression of BAG3 could improve the lipid profile and suppress atherosclerosis in ApoE^−/−^ mice. Moreover, overexpression of BAG3 in HUVECs was found to stimulate autophagy, and this effect was reversed after an introduction of an inhibitor of ATPase, HSP70 [89]. However, it should be remembered that the activity of HSP70 is associated with the suppression of classical autophagy [90]. Mechanisms induced by HSP60 and HSP70 in the context of atherosclerosis are summarised in Figure 2.

HSP90 represents another member of the HSP family. The protein exerts numerous functions due to its possibility to bind co-chaperones. HSP90 has multiple co-chaperone binding sites; together, they form symmetric and asymmetric complexes, which impact HSP90 functionality [91]. Current evidence highlights the potential of HSP90 to regulate the progression of atherosclerosis as well. Researchers have shown that extracellular HSP90α enhances endothelial cell inflammatory responses [92].

S-nitrosilation (SNO) is one of the post-translational modifications of HSP90, which alters the activity of ATPase and has been suggested to influence endothelial NO synthase [91]. In endothelial cells stimulated with oxLDL and in atherosclerosis samples, an increased presence of SNO-HSP90 is observed. Due to the SNO modification, limited access to NO, together with an enhanced inflammatory response, is observed in endothelial cells, thus pointing to endothelial dysfunction [93]. Moreover, HSP90 has been suggested as a potential marker of atherosclerosis [92]. In the literature, one can find significant evidence of HSP90 inhibitors. However, these studies mainly refer to oncological contexts. Nevertheless, studies also describe their anti-inflammatory properties [94,95,96], which could be investigated in atherosclerosis in the future.

Overall, different members of the HSP family affect cells implicated in the pathogenesis of atherosclerosis. Several interesting mechanisms should be mentioned when discussing the involvement of HSPs in pathophysiology, including autoimmune reactions, immunisation, inflammation, and autophagy, as well as in the potential use of HSP inhibitors. Importantly, cells seem to be able to modify the expression of HSPs to prevent the progression of this vascular disorder.

## 3. Alarmins and Myocardial Infarction

### 3.1. HMGB1

Myocardial infarction (MI) is a disease that is associated with permanent damage to the heart muscle, which causes the death of cells as a result of prolonged ischaemia of the heart [97,98]. The main risk factors for MI are significant body weakness due to complications from CVDs—including coronary artery disease, atherosclerosis, coronary artery injury—and coronary wall thickening due to metabolic disease or hyperplasia (Fabry disease) [99]. Other important MI risk factors include spasms that cause a narrowing of the blood vessels, spontaneous cleavage of the coronary arteries, obesity, a sedentary lifestyle, smoking, diabetes, and elevated blood cholesterol [99,100]. Every year, more than 7 million people are diagnosed with an MI, including in developed and developing countries, making it one of the leading causes of death worldwide [100]. Men over 60 are most likely to suffer from the disease [101]. MI induces a process of heart remodelling that frequently leads to heart failure, followed by a healing process that is coordinated by the immune system. This mechanism works to repair and modify damaged tissues following MI [98,102]. The healing process may contribute to the effects of progressive heart disease by supporting the death of cardiomyocytes, but it can also promote regenerative processes that lead to reduced inflammation [102,103]. Several stages can be distinguished in this process: the inflammatory phase (covering the first 72 h following the infarction), the healing phase (7–10 days following the infarction) and the remodelling phase (lasting up to several months). A prolonged state of ischaemia stimulates mechanisms that are responsible for the deaths of cardiomyocytes and parenchyma cells, which are mainly caused by necrosis, derivative apoptosis, or autophagy. Necrotic and defective cells consequently release intracellular matter and DAMPs, which trigger an immune response after binding to PRRs [102,103]. Moreover, DAMPs may be released by cardiomyocytes and fibroblasts under conditions of enhanced oxidative stress [103]. Various molecules belong to the DAMP family, including HMGB1, heat shock proteins, S100, ATP, mitochondrial DNA, dsRNA, and ssRNA, in addition to interleukins that interact mainly with TLR. As a result of ligand–receptor coupling, non-specific-response cells such as neutrophils, monocytes/macrophages, eosinophils, and dendritic cells (DCs) are attracted to the infarction zone [98]. During the initial phase of inflammation, neutrophils generate a large amount of ROS and proteases, which intensify the damage to vessels and tissues within the infarction. In contrast, monocytes rapidly transform into macrophages that are responsible for the secretion of cytokines such as IL-1, IL-6, and TNFα, which initiate an acute inflammatory response [98,104,105]. Eosinophils support the transformation process by secreting IL-4, and are thus responsible for limiting the adhesion of neutrophils to the endothelial wall [102]. In the healing phase, macrophages and neutrophils eliminate cellular debris and prevent digestion of the ECM. Simultaneously, macrophages undergo a phenotypic transformation towards the anti-inflammatory variants that are associated with the initiation of angiogenesis and the process of collagen creation through the secretion of anti-inflammatory cytokines, including IL-4, IL-10, and IL-13 [98,106,107]. During the phase of remodelling, an important role is played by lymphocytes that migrate to the region of the infarction. CD4+ cells are the most abundant type of T cells that are present after MI, and they take part in myocardial healing and the conversion of monocytes into macrophages. In contrast, B lymphocytes—with the help of CCL7, which they secrete—are responsible for the increased infiltration of pro-inflammatory monocytes that leads to cardiac dysfunction [98,107]. Moreover, B cells contribute to post-MI atherosclerosis progression, as a depletion of these lymphocytes could reduce size of the pathological vascular lesion. The effect of B cells on atherosclerosis progression was suggested to be mediated by alarmins, which links both MI and atherosclerosis [108].

HMGB1 is a crucial factor in MI, as cardiomyocytes have been shown to passively secrete it in vivo in response to oxidative stress and peroxygen necrosis. In addition, HMGB1 is released as a consequence of damage to the ischaemia–reperfusion (IR) heart muscle [109,110]. Immediately after its secretion by cells, HMGB1 migrates by diffusion to the peri-infarction region, where it activates specific TLR receptors (TLR2, TLR4, TLR9) and RAGE, all of which induce neutrophils, monocytes, and macrophages to release pro-inflammatory cytokines, including TNFα, IL-1, IL-6, and IL-8 [103,109,111]. Among the cardiomyocyte receptors found in the heart muscle, the most sensitive type is the TLR4 receptor; its activation results in the increased expression of CXC-type chemokines and adhesion molecules, such as intercellular adhesion molecule 1 (ICAM-1) and intercellular adhesion molecule 2 (VCAM-2), which are responsible for directing monocytes to the infarction zone. Subsequently, these cells secrete a range of cytokines—including TNFα, IFNγ, IL-1-2, IL4-6, IL-10, IL-17, and granulocyte macrophage colony-stimulating factor (GM-CSF)—that influence the proteins of the ECM by affecting the reorganisation process [109,112]. This pathway also involves the recruitment of neutrophils and the phenotypic transformation of macrophages [113,114]. Karuppagounder et al. have shown that the transformation of macrophages by HMGB1 is associated with the ageing process of the heart. Research on SAMP80 mice, which are susceptible to accelerated ageing, showed that these SAMP80 mice had fewer anti-inflammatory macrophages in the heart than the controls did. This phenomenon is associated with reduced concentrations of the specific markers CD36 and IL-10 [115]. In contrast, Oyama et al. presented results indicating that a deficiency in TLR4 signalling significantly reduces the occurrence of MI after a period of ischaemia by reducing the expression of chemokines, the infiltration of PMN, and the formation of ROS [112]. The second type of signalling by HMGB1 is RAGE-based signalling, which is partly related to the activation of MAPK and NF-κB pathways; these cascades mobilise leukocytes that increase the size of the infarction through even greater tissue damage and cell apoptosis [103,113]. However, studies also indicate that in the later phases, HMGB1 takes part in the regeneration of the heart. In an in vivo experiment, Kitahara et al. discovered that HMBG1 released from dead mouse cardiomyocytes enhanced angiogenesis through autocrine and paracrine mechanisms, thereby restoring heart function and increasing the probability of survival [116]. Limana and others proved via experiments conducted on mice that the introduction of HMGB1 into the left ventricle (LV) of the heart immediately after an infarction promotes muscle regeneration by activating the proliferation and differentiation of c-kit+ cells; during acute MI, c-kit+ cells were transformed into cardiomyocytes [117]. Following MI, plasma concentrations of HMGB1 increase rapidly in both humans and rats. In rodents, HMGB1 expression increases within 2–3 days depending on the species [113,118]. Andrassy et al. demonstrated, via magnetic resonance imaging, that HMGB1 expression is associated with infarction size for STEMI area under curve (AUC) = 0.93, (95% CI = 0.85–0.98) and NSTEMI AUC = 0.96 (95% CI = 0.86–0.99) [119]. Significantly increased levels of HMGB1 in patients diagnosed with acute coronary syndrome cause a decreased heart rate and negative LV remodelling [118]. However, following coronary blockage in rats and the introduction of HMGB1, cardiac function was improved via the modulation of inflammation by reducing DC accumulation [120]. In their studies, Kohno et al. showed that anti-HMGB1 treatment affects the abnormal reconstruction of the left heart ventricle but protects it from the accumulation of macrophages and higher concentrations of TNFα and IL-1β [118]. On the other hand, a local injection of exogenous alarmin improves cardiac function [117]. The systemic introduction of anti-HMGB1 improves the function of a heart that has undergone IR [113]. Experiments have shown that the introduction of ethyl pyruvate into the body blocks the secretion of HMGB1, which supports the maintenance of heart function after long-lasting cardiac ischaemia [121]. Despite the fact that much is known about alarmin, it is difficult to unequivocally state whether it is a positive or a negative factor in the treatment of MI. Hence, methods for the use of alarmins in clinical treatment are yet to be developed.

### 3.2. Heat Shock Proteins

The heat shock protein family of molecules also induces immune responses in MI. Several proteins stand out as being involved in the response to this pathological condition. One of these is HSP60, which is released by damaged cardiomyocytes, but its precise mechanism of action has not yet been fully confirmed. HSP60 is found in both the plasma and the extracellular space of cardiomyocytes [122,123]. In the plasma, the TLR-4 receptors stimulate the production of cytokines, primarily TNFα [111,122,124]. What is more, HSP60 may affect monocytes and stimulate the complement system [122]. Studies conducted on rats by Li et al. have shown that apoptosis initiated by HSP60 is directly bound by TLR-4 and NF-κB. The introduction of anti-HSP60 or TLR-4 antibodies with deletion into the rodent body caused them to significantly reduce IR [125]. There is a form of extracellular HSP60 (exHSP60) that is released by the exosomes of cardiomyocytes. ExHSP60 binds to the TLR4 receptor, which affects the production of TNFα and IL-6, as has been confirmed by experiments conducted in rats [124]. In addition, this protein may generate myocyte apoptosis and contribute to increased heart failure [124,126]. Another protein that increases in amount after a heart attack is HSP70, but its role is not fully understood. It has been shown that intracellular HSP70 positively affects the heart by initiating processes related to blocking apoptosis, reducing oxidative stress, and correcting endothelial function [127,128]. HSP70 overexpression in Sprague Dawley rats favoured arteries and capillaries [129]. In contrast, therapy with lipopolysaccharide (LPS) caused HSP70 to inhibit the NF-κB pathway, and this subsequently reduced oxidative stress and inflammation and inhibited apoptosis following a blockage of the left anterior descending coronary artery (LAD) in rats [130]. A particular version of HSP70 is HSP72; the in vivo overexpression of HSP72 in transgenic mice contributed to a reduced infarction size due to ischaemia and cardiac reperfusion [131]. Other studies conducted in rabbits have shown that the administration of a single intravenous dose of HSP72 inhibited myocyte apoptosis and restored left ventricular function that had been destroyed in IR [131]. In contrast, exHSP70, which is secreted by exosomes of cardiomyocytes, negatively affects the heart during infarction. ExHSP binds to TLR2/TLR4 and activates CD14-dependent mechanisms by stimulating monocytes to produce pro-inflammatory cytokines such as TNFα, IL-1β, and IL-6 [126]. Interestingly, elevated HSP70 levels positively affected infarction size in rats with previously bound coronary vessels that had been orally administered bimoclomol. However, this study is not clinically significant because the agent was administered at different times prior to the infarction itself [132]. HSP27 is a protein that exhibits prophylactic effects on the heart after MI [131]; overexpression of this protein contributes to securing the integrity of the microtubules and cytoskeleton of cardiac myocytes [133]. It is likely that HSP37 binds to AKT, which consequently triggers an anti-apoptotic mechanism [134].

### 3.3. S100 Proteins

The S100 family of proteins is involved in the pathogenesis of MI. The induction of MI observed in a mouse model led to the rapid recruitment of neutrophils to the sites of infarction foci, where S100A8 and S100A9 alarmins were released. Subsequently, the binding of S100A8 and S100A9 to TLR4 led to the activation of the caspase-activating complex known as the NLRP3 inflammasome, which is accompanied by the release of pro-inflammatory IL-1β from naïve neutrophils. IL-1β interacts with Interleukin 1 Receptor Type 1 (IL-1R1) receptors—which are located on haematopoietic stem cells and progenitor cells in the bone marrow—to stimulate granulopoiesis [135]. Furthermore, it has been suggested that suppression of the S100A8/A9 pathway and the granulopoiesis that follows can improve cardiac function after MI. Rather than directly promoting neutrophil degranulation, S100A9 primarily promoted neutrophil and macrophage recruitment, thus resulting in infarct wall thinning in a mouse model [136]. Importantly, Li et al. [137] demonstrated that by acting as calcium-binding proteins, S100A8/A9 are key regulators of cardiomyocyte mortality, whereas haematopoietic overexpression of S100A9 leads to myocardial ischaemia–reperfusion injury through the inhibition of mitochondrial function. Impaired mitochondrial function resulted in defective respiratory chain function and a reduced ATP pool, leading to significant cellular damage and cardiomyocyte apoptosis [138]. Furthermore, the administration of a S100A9-neutralising antibody significantly reduced MI/R injury and improved myocardial function, thereby providing evidence of a targeted pharmacological strategy. The short-term blockade of S100A9 by small-molecule drugs clearly demonstrated a reduced inflammatory response and improved myocardial function in mouse models [139]. Furthermore, Boteanu et al. [140] reported that this pharmacological strategy upregulated the expression of proteins involved in leukocyte recruitment, which resulted in more efficient repair of cardiomyocytes after ischaemia; reductions in cardiac stress markers, including NPM1, TPT1, and NOL3; and the beneficial regulation of anti-apoptotic and propapotic proteins. Specifically, upregulated NPM1 expression was observed in the peripheral blood of patients with MI, which was significantly positively correlated with adverse prognostic indicators of MI. By downregulated TSC1 expression and thereby increasing mTOR pathway activation in macrophages [141], S100A8 and S100A9 regulate the autophagy and apoptosis of cardiomyocytes through multiple signalling pathways. However, they play major roles in early MI through the MAPK and PI3K signalling pathways by significantly affecting the expression of Bcl and Casp9 [142]. During ischaemia, Bcl-2 production is inhibited; this enhances the synthesis of BAX and Casp9, which activates Casp3, allowing it to activate the final stage of the mitochondrial pathway of apoptosis [143]. The long-term inhibition of S100A9 leads to accelerated left ventricular remodelling with a decreased ejection fraction (EF) and fractional shortening, thus leading to the progressive degradation of myocardial function [144]. This is due to a reduction in the number of repair macrophages, which results in the impaired removal of apoptotic cardiomyocytes, subsequently causing cardiac remodelling with impaired LV function. In addition, microRNA-24 is downregulated, which downregulates S100A8 expression and consequently inhibits the TLR4/MyD88/NF-κB pathway, thereby suppressing inflammatory cell infiltration in the infarcted area [145]. Another important small calcium-binding protein that may affect the regulation of cardiac function is S100A1. Reduced levels of this protein in mice have been shown to reduce endothelium-dependent vasorelaxation and lead to increased systemic arterial pressure, reduced coronary reserve, and increased mortality after MI [146]. Fan et al. [147] demonstrated that the plasma S100A1 level was significantly higher in patients with STEMI than in patients with coronary artery disease. A significant positive correlation between S100A1 and a marker of cardiomyocyte damage (troponin) was observed in addition to a negative correlation between S100A1 and cardiac functionality left ventricular ejection fraction (LVEF). Furthermore, alarmin levels were positively correlated with those of less-specific CK-MB and NT-proBNT markers, as well as with the inflammatory marker CRP. Moreover, the authors suggested the use of S100A1 as a potential cardiac biomarker in STEMI (AUC = 0.87, 95% CI: 0.84–0.91). The confirmation of the potential clinical use of the above biomarker in acute MI (AMI) is also confirmed by a study [148] in which a chemiluminescent immunoassay for the determination of S100A1 was developed (AUC = 0.7779, 95% CI: 0.6812–0.8745). The modified increase in the expression of S100A6 weaken apoptosis, reduces myocardial hypertrophy and infarct focus size, but the exact mechanism is not yet well understood. This leads to a reduction in myocardial ischaemia–reperfusion, which may result in a higher survival rate in patients following myocardial ischaemia–reperfusion [149]. Another protein that may have a cardioprotective function is S100A4. Doroudgar et al. [150] show that increased expression of S100A4 protects cardiomyocytes after MI by promoting PI3K/AKT signalling and improving myocardial function. PI3K class 1, via the phosphorylation of PIP2, contributes to the formation of PIP3 binding AKT, which activates proliferation, survival, and metabolic regulation. In addition, PI3K class 1 is responsible for the regulation of voltage-gated Ca^2+^ channels in cardiac muscle and VSMCs promoting Ca^2+^ influx into the cell. This mechanism can be used in targeted pharmacotherapy [151]. In addition, dual regulation of S100A4 in mice reduces cardiac fibrosis after MI through the Wnt/β-catenin regulatory pathway [152]. However, one of the Wnt proteins, Wnt-5a, has pro-inflammatory effects by releasing IL-1, IL-6, and IL-8 from monocytes, thereby activating β-catenin pathways located in pro-inflammatory macrophages, which are activated after MI; this leads to the increased infiltration of inflammatory cells and increased inflammation. In turn, an increase in S100A4 expression may inhibit β-catenin pathway signalling and suppress inflammation, thus providing a potential therapeutic target [153].

### 3.4. Interleukin-33

IL-33 is an important regulator of the immune response. Studies in both animals and humans have highlighted the cardioprotective function of IL-33/ST2. IL-33 activates the key IL-33/JAK-STAT/M2 pathway that is involved in myocardial remodelling after MI. Specifically, it has been shown to promote the presence of cardiac M2 macrophages in the infarcted area, thus suppressing the early inflammatory response and consequently, ventricular remodelling [154]. In addition, the systemic deletion of IL-33 in mice triggers an enhanced inflammatory response directed at cardiomyocytes, which leads to myocardial hypertrophy and fibrosis and results in impaired myocardial contractility [155]. Studies have proposed the use of the sST2 receptor as biomarker in heart failure, but conflicting results have been published. In a review by Sciatti et al., the authors investigate the use of sST2 as a potential predictor of prognosis in acute and chronic heart failure, while highlighting the lack of validation of this biomarker and modifications by some drug groups, such as SGLT2i [156]. However, a study of IL-33 levels in patients with acute coronary syndrome (ACS) showed that IL-33 levels gradually decreased after ACS in patients with ST-segment elevation myocardial infarction (STEMI), in contrast with sST2, which has constant levels over the same period. Furthermore, the AUC for IL-33 was 0.826 (95% CI = 0.689–0.962; *p* < 0.001) [157]. In addition, the measurement of IL-33 and sST2 serum levels has shown promising diagnostic efficacy as a marker of adverse cardiovascular events following percutaneous coronary intervention (PCI); the AUCs of these molecules were 0.675 and 0.872, respectively [158].

Targeting a reduction in the signalling of IL-33/ST2 in order to reduce infarct size and improve cardiac function is possible in mouse models, as was demonstrated by Chen and colleagues [159]. The use of beta-blocker therapy after MI enhanced the signalling of this pathway by decreasing sST2 expression, which showed no effect on IL-33 upregulation. Many authors point to the enhancement of the IL-33/ST2 signalling pathway and improvements in LV function via increasing sST2 levels—which is achieved by the use of eplerenone [160] and other mineralocorticoid receptor antagonists (MRAs) such as spironolactone after MI—without any significant effects on IL-33 levels [161]. Interestingly, Lax et al. demonstrated that the use of MRAs in rats that had not undergone MI led to an increase in cardioprotective IL-33 levels.

## 4. Conclusions

In conclusion, alarmins are implicated in a broad number of interactions that regulate inflammatory responses. In patients and animal models with CVDs, alarmin serum concentration or alarmin expression in affected tissues is altered, suggesting that these molecules are somehow implicated in the pathogenesis of the disease. Atherosclerosis is associated with a greater presence of HMGB1. Current evidence demonstrates that the pro-inflammatory responses induced by HMGB1 take part in the enhancement of inflammatory responses, as it stimulates the secretion of pro-inflammatory cytokines. Moreover, as silencing of HMGB1 has been associated with beneficial outcomes, it might represent an important therapeutic target in the future. In myocardial infarction, HMGB1 seems to both induce inflammatory reactions, which could increase tissue damage, and myocardial regeneration by enhancing angiogenesis. Moreover, elevated expression of S100A8/A9 in atherosclerotic lesions could imply the greater presence of neutrophils and plaque instability. However, controversial results obtained regarding the role of IL-33 may suggest that the involvement of alarmins under pathological conditions can depend on the cellular context of methods. In addition, future studies should further investigate HSP60 immunisation and the role of co-chaperones in the pathogenesis of atherosclerosis. Overall, several mechanisms stimulated by alarmins have been recognised over the years. However, further investigations are required to understand the implications of these molecules in CVDs and use them as diagnostic biomarkers or therapeutic targets. Intriguingly, alarmins were suggested to act as atherosclerosis residual risk markers [162], which should also be further explored.

## Figures and Tables

**Table 1:** Summary of impact of HMGB1 on VSMCs and endothelial cells that are associated with atherosclerosis.

Cell Type	Mechanism Linking to Pathogenesis of Atherosclerosis	References
Vascular smooth muscle cells	HMGB1 enhances the expression of CRP and MMP-2. HMGB1 is involved in a signalling loop with NLRP3 inflammasome, as it enhances its expression. Furthermore, the inflammasome stimulates the formation of VSMC foam cells. Silencing HMGB1 potentiates the anti-inflammatory properties of other agents to suppress the synthetic phenotype of VSMC. HMGB1 is involved in the vascular calcification process. Inflammation and migration of VSMCs can be targeted using miRNAs miR-141-5p, miR-129-5p, miR-34c, and miR-24.	[18,25,26,28,31,32,33,34]
Endothelial cells	Ox-LDL enhances HMGB1 expression and cytoplasm translocation. Silencing HMGB1 reduces endothelial cell damage induced by modified LDL. HMGB1 enhances endoplasmic reticulum stress in endothelial cells.	[48,54]
